# Gender Role Orientation with Health Literacy and Self-Efficacy for Healthy Eating among Japanese Workers in Early Adulthood

**DOI:** 10.3389/fnut.2016.00017

**Published:** 2016-06-08

**Authors:** Chizuru Hosokawa, Hirono Ishikawa, Masafumi Okada, Mio Kato, Tsuyoshi Okuhara, Takahiro Kiuchi

**Affiliations:** ^1^Department of Health Communication, School of Public Health, Graduate School of Medicine, The University of Tokyo, Tokyo, Japan

**Keywords:** healthy eating, gender role orientation, gender norms, health literacy, self-efficacy, androgynous

## Abstract

Gender role, independent of biological sex, affects health. However, research on healthy eating that considers the importance of gender norms is scarce. People who are androgynous and have high masculinity and femininity are reported to have better health practices than other people. The present study aimed to examine the differences in health literacy (HL) and self-efficacy for healthy eating by gender role in Japanese men and women. Participants were 629 men and women aged 25–34 years, recruited via a Japanese Internet research company database. Participants were categorized into four gender role groups using the Japanese Gender Role Index. HL and self-efficacy for healthy eating were assessed using the healthy eating literacy (HEL) scale and the healthy eating and weight self-efficacy (HEWSE) scale. Analysis of variance with Bonferroni-adjusted *post hoc* tests and hierarchical multiple regression were used to test the research hypotheses. We found that the Androgynous group had significantly higher HEL and HEWSE scores than the Feminine and Undifferentiated groups. The Masculine group scored significantly higher on both measures than the Undifferentiated group. Being Androgynous (HEL: β = 0.34, *p* < 0.001; HEWSE: β = 0.30, *p* < 0.001) was a strong predictor for higher scores even after considering other predictors. The results showed significant associations between gender role orientation and individual HL and self-efficacy for healthy eating. These findings may be relevant for promoting healthy eating from the perspective of gender norms.

## Introduction

In general, compared with women, men lack knowledge and skills about food and nutrition, and less likely to engage in food preparation (Ministry of Health, Labour and Welfare, 1999, 2000). It has been suggested that such differences might contribute to less healthy eating behaviors and lower health status among men (1–3). However, several studies have emphasized that gender role, independent of biological sex, affects health (4–6). Unlike biological sex, gender norms can be changed by educational approaches to promote healthy eating. But there have been little research on gender norms and healthy eating.

Gender role orientation represents a person’s position within the framework of masculine and feminine dimensions (7). A measurement scale for gender role orientation has been developed in the field of psychology. The Bem Sex Role Inventory (BSRI: 1974) is a commonly used gender role orientation scale in many countries (4). Studies suggested that people who are androgynous (i.e., have high masculine as well as feminine traits) have flexibility in behaviors depending on the situational appropriateness (8), and have better health practices than those with other gender role orientation (9). On the other hand, undifferentiated people who are low in both masculine and feminine traits have fewer behavior options (10), and are more likely to take risky behaviors (9). In Japan, it has been reported that people who are androgynous have higher self-esteem and better psychosocial adjustment (11), and middle-aged androgynous women had higher quality of life scores (12). However, research in this area is still limited in Japan. Research on dietary behaviors that considers the effect of gender and gender norms is particularly scarce (13).

In addition, health literacy (HL) and self-efficacy are meaningful outcomes to explore in relation to gender role. It has recently been suggested that HL about diet is integral to increasing dietary knowledge and promoting healthy dietary habits (10). In Japan, the Ministry of Health, Labour and Welfare emphasized the necessity of providing “access to information” about food to practice an appropriate diet. Self-efficacy is the conviction that one can successfully execute the behavior required to produce the outcomes (14). It also has an important role in healthy eating behaviors (15). However, there have been few studies exploring the effect of gender role on these outcomes.

It has been suggested that by early adulthood, people tend to have a fixed gender role, and fit their lifestyles and behaviors to it (16, 17). Early adulthood is the time between adolescence and middle age that is roughly defined as between the ages 20 and 40 years. Although healthy eating is important for everyone to maintain their health, studies have found that people who adopted healthy dietary patterns in early adulthood were healthier in middle age (18, 19). Therefore, early adulthood is an appropriate age group in which to investigate the relationship between gender role orientation and food-related beliefs and attitudes.

The present study aimed to examine the relationship between gender role orientation and HL and self-efficacy for healthy eating among Japanese in early adulthood. More specifically, two hypotheses were examined:

1) Among gender role orientation groups, those who are androgynous have the highest scores in both HL and self-efficacy, while those with an undifferentiated gender role have the lowest scores.
2) Gender role orientation would better account for individual differences in HL and self-efficacy scores than biological sex.

## Materials and Methods

### Participants

Data for the present study were collected in September 2015, via an online survey from 629 men and women who were registered with a Japanese Internet research company database. As part-time workers and homemakers have different lifestyles from full-time workers, full-time workers were selected for the purpose of this study. In addition, white- and blue-collar workers might differ in educational attainment, income, and health status (20, 21). These factors are associated with HL, self-efficacy (16), and gender role orientation (22, 23). Therefore, we recruited white-collar workers aged 25–34 years (20s and 30s the same number), and the participants were stratified by sex (male and female the same number). This study was approved by the Ethical Committee of the University of Tokyo on August 5, 2015 (No. 10901).

### Measurements

#### Gender Role Orientation

Japan has developed in significantly different ways from Western countries, which has influenced gender role development in Japanese people. Western measures of gender roles, such as the BSRI, are not relevant for Japanese. For example, Japanese people saw masculine stereotypes as less active than did people in other countries (22). The Japanese Gender Role Index (JGRI) is a gender personality scale for the Japanese population developed in 2002, and consists of two subscales: a 10-item femininity scale (F) and a 10-item masculinity scale (M). Similar to the BSRI, the JGRI uses adjectives that describe socially desirable masculine/feminine characteristics in Japanese society. Participants were asked to rate their personal quality for each of the JGRI items on a 7-point scale. Participants were categorized into four gender role groups using the median split method. Participants with high scores on both subscales were classified as “Androgynous.” Those who scored high on F and low on M were categorized as “Feminine”; high on M and low on F were “Masculine”; and those with a low score on both subscales were categorized as “Undifferentiated.” The scores for the items on each subscale were summed and divided by the number of items in the scale to yield a subscale score. The possible range of the subscale scores is between 1 and 7. The internal consistency (Cronbach’s α coefficient) was 0.86 for F and 0.93 for M. Items were presented in a randomized order using an online survey program.

#### Healthy Eating literacy

Literacy about healthy eating was assessed with the healthy eating literacy (HEL) scale (23). The HEL consists of five items about food-related factors, and is validated with the transtheoretical model of healthy diet (24). The HEL is a content-specific version of the HL scale (25) for food-related information. The subscale scores range from 1 (strongly disagree) to 5 (strongly agree). The internal consistency of the HEL was 0.86.

#### Healthy Eating and Weight Self-Efficacy

Self-efficacy for healthy eating and weight was assessed by the healthy eating and weight self-efficacy (HEWSE) scale (15). The HEWSE consists of two domains: consumption of healthy foods (seven items) and healthy weight (four items). Participants rated their beliefs about their ability to engage in healthy eating and weight maintenance behaviors on a 5-point Likert scale ranging from 1 (strongly disagree) to 5 (strongly agree). The internal consistency of the HEWSE was 0.89.

#### Food-Related Experiences

As everyday food preparation or food-related education may influence individual HL or self-efficacy for healthy eating, we asked about participants’ food-related experiences. This included meal preparation frequency, “*How often do you prepare (cook) your meal (breakfast/lunch/dinner)?*” Response options were: 1 = never; 2 = less than once a month; 3 = a few times per month; 4 = 1–2 times per week; 5 = 3–4 times per week; 6 = almost every day. Activities, such as pouring hot water or milk, or buying something to eat, were not counted as food preparation. Considering the lifestyles of full-time workers, we used dinner to assess frequency of meal preparation. We also asked whether or not participants had engaged in food education, such as education about nutrition/cooking in an educational institution/hospital.

#### Sociodemographic and Other Factors

We collected information on age, sex, marital status, body mass index (BMI: weight in kilogram/height in square meter), self-perceived weight status (too heavy, somewhat heavy, just about right, somewhat light, or too light), educational level, occupation, and annual household income.

### Analytical Method

Previous studies refer to the effectiveness of median split method to disentangle health effects of each group (4), we used the median split method to separate individuals into four groups. Then, distributions of sociodemographic variables across sex and gender role were compared using *t*-tests and χ^2^ tests. Analysis of variance (ANOVA) with Bonferroni-adjusted *post hoc* tests were used to test for differences in HEL and HEWSE scores by gender role groups. Hierarchical multiple regression was used to test the research hypotheses. The dependent variables were HEL and HEWSE scores. Participants’ age, sex, marital status, educational level, annual household income, and self-perceived weight status were entered in the first step of the regression analysis. Participants’ gender role orientation was entered in the second step, and food-related experiences were entered in the final step. Frequency of dinner preparation was categorized as: “Never”; “Sometimes” (less than once a month, a few times per month, 1–2 times per week); and “Often” (3–4 times per week, almost every day). The analyses were performed using R Statistical Software (version 3.1.2; R Foundation for Statistical Computing, Vienna, Austria).

## Results

### Participants’ Characteristics

Table 1 shows the basic characteristics of the study participants. The mean HEL score was 3.37 [standard deviation (SD) = 0.77] and the mean HEWSE score was 3.19 (SD = 0.69). There were no significant sex differences in HEL and HEWSE scores. Compared with women, men had significantly higher educational attainment (*p* < 0.001), higher income status (*p* < 0.01), were more likely to be married (*p* < 0.001), had higher BMI (*p* < 0.001), and had lower frequency of dinner preparation (*p* < 0.001).

**Table 1:** **Basic characteristics of study participants by sex (*n* = 629)**.

	Men (*n* = 313) *n* (%)	Women (*n* = 316) *n* (%)	Total (*n* = 629) *n* (%)	*p*-value
HEL (mean ± SD)	3.34 ± 0.80	3.41 ± 0.75	3.37 ± 0.77	0.21^a^
HEWSE (mean ± SD)	3.19 ± 0.70	3.19 ± 0.67	3.19 ± 0.69	0.96^a^
Age (mean ± SD, years)	30.5 ± 2.6	29.5 ± 2.8	30.0 ± 2.8	
Gender type				0.02^b^
Androgynous	126 (40.3)	104 (32.9)	230 (36.6)	
Feminine	37 (11.8)	42 (13.3)	79 (12.6)	
Masculine	40 (12.7)	31 (9.8)	71 (11.3)	
Undifferentiated	110 (35.1)	139 (44.0)	249 (39.6)	
Education				<0.001^b^
University	221 (70.6)	172 (54.4)	393 (62.5)	
Less than university	92 (29.4)	144 (45.6)	236 (37.5)	
Body mass index (mean ± SD, kg/m^2^)	22.3 ± 3.4	20.3 ± 3.4	21.3 ± 3.5	<0.001^b^
<18.5	25 (8.0)	82 (25.9)	107 (17.0)	
18.5-24.9	232 (74.1)	213 (67.4)	445 (70.7)	
≥25.0	55 (17.6)	21 (6.6)	76 (12.1)	
Self-perceived weight status				0.52^b^
Too heavy, Somewhat heavy	113 (36.1)	128 (40.5)	241 (38.3)	
Just about right	127 (40.6)	118 (37.3)	245 (39.0)	
Too light, Somewhat light	73 (23.3)	70 (22.2)	143 (22.7)	
Marital Status				<0.001^b^
Married	161 (51.4)	108 (34.2)	269 (42.8)	
Not married	152 (48.6)	208 (65.8)	360 (57.2)	
Occupation				<0.001^b^
Administrative and managerial workers	13 (4.2)	2 (0.6)	15 (2.4)	
Professional and engineering workers	105 (33.5)	75 (23.7)	180 (28.6)	
Clerical workers	93 (29.7)	159 (50.3)	252 (40.1)	
Sales workers	39 (12.4)	21 (6.6)	60 (9.5)	
Service workers	53 (16.9)	59 (18.7)	112 (17.8)	
Security workers	10 (3.2)	0 (0)	10 (1.6)	
Income quintile				0.002^b^
~3,999,999	85 (27.2)	114 (36.1)	199 (31.6)	
4,000,000~5,999,999	111 (35.5)	78 (24.7)	189 (30.0)	
6,000,000~9,999,999	95 (30.4)	85 (26.9)	180 (28.6)	
~10,000,000	22 (7.0)	39 (12.3)	61 (9.7)	
Dinner preparation frequency				<0.001^b^
Never	83 (26.5)	34 (10.8)	106 (16.9)	
Sometimes	148 (47.3)	103 (32.6)	251 (39.9)	
Often	82 (26.2)	179 (56.6)	261 (41.5)	
Engaged in food education				0.23^b^
Yes	24 (7.7)	34 (10.8)	58 (9.2)	
No	289 (92.3)	282 (89.2)	571 (90.8)	

*^a^*t*-test*.

*^b^Chi-square test*.

*Dinner preparing frequency was categorized by Never: never; Sometimes: less than once a month, a few times per month, 1–2 times per week; Often: 3–4 times per week, almost every day. Engaged in food education contains education regarding nutrition/cooking in an educational institution/hospital*.

The mean scores for the JGRI subscales were F = 3.54 (SD = 1.02) and M = 3.49 (SD = 1.35). There was no significant difference in JGRI scores between the sexes. The median scores for both subscales were 3.40. In total, 37% of participants were categorized as Androgynous, 13% as Feminine, 11% as Masculine, and 40% as Undifferentiated. This indicated that there were relatively few gender-typed individuals (Feminine and Masculine). While the percentages of men in the Androgynous group and women in the Undifferentiated group were high (androgynous 40 vs. 33%; undifferentiated 35 vs. 44% for men and women, respectively), no significant difference was observed in gender role distribution by biological sex (χ^2^ = 6.93, df = 3, *p* = 0.07). Across the four gender role groups, there was a significant difference in educational attainment (Table 2).

**Table 2:** **Basic characteristics of study participants by gender role orientation (*n* = 629)**.

	Androgynous (*n* = 230) *n* (%)	Feminine (*n* = 79) *n* (%)	Masculine (*n* = 71) *n* (%)	Undifferentiated (*n* = 249) *n* (%)	Total (*n* = 629) *n* (%)	*p*-value^a^
Age (mean ± SD, years)	29.6 ± 2.8	30.1 ± 2.8	30.3 ± 2.6	30.1 ± 2.8	30.0 ± 2.8	
Sex						0.07
Men	126 (54.8)	37 (46.8)	40 (56.3)	110 (44.2)	313 (49.8)	
Women	104 (45.2)	42 (53.2)	31 (43.7)	139 (55.8)	316 (50.2)	
Education						<0.001
University	143 (62.2)	22 (27.8)	21 (29.6)	155 (62.2)	236 (37.5)	
Less than university	87 (37.8)	57 (72.2)	50 (70.4)	94 (37.8)	393 (62.5)	
Body mass index (kg/m^2^)						0.94
<18.5	40 (17.4)	15 (19.0)	10 (14.1)	42 (16.9)	107 (17.0)	
18.5–24.9	166 (72.2)	53 (67.1)	52 (73.2)	174 (69.9)	445 (70.7)	
≥25.0	24 (10.4)	11 (13.9)	9 (12.7)	32 (12.9)	76 (12.1)	
Self-perceived weight status						0.14
Too heavy, somewhat heavy	76 (33.0)	32 (40.5)	24 (33.8)	109 (43.4)	241 (38.3)	
Just about right	104 (45.2)	31 (39.2)	27 (38.0)	83 (33.3)	245 (29.0)	
Too light, somewhat light	50 (21.7)	16 (20.3)	20 (28.2)	57 (22.9)	143 (22.7)	
Marital Status						0.15
Married	106 (46.1)	35 (44.3)	35 (49.3)	93 (37.3)	269 (42.8)	
Not married	124 (53.9)	44 (55.7)	36 (50.7)	156 (62.7)	360 (57.2)	
Occupation						
Administrative and managerial workers	8 (3.5)	4 (5.1)	1 (1.4)	2 (0.8)	15 (2.4)	
Professional and engineering workers	69 (30.0)	20 (25.3)	21 (29.6)	70 (28.1)	180 (28.6)	
Clerical workers	82 (35.7)	34 (43.0)	28 (39.4)	108 (43.4)	252 (40.1)	
Sales workers	24 (10.4)	5 (6.3)	9 (12.7)	22 (8.8)	60 (9.5)	
Service workers	44 (19.1)	14 (17.7)	9 (12.7)	45 (18.1)	112 (17.8)	
Security workers	3 (1.3)	2 (2.5)	3 (4.2)	2 (0.8)	10 (1.6)	
Income quintile						0.21
~3,999,999	70 (30.4)	29 (36.7)	13 (18.3)	87 (34.9)	199 (31.6)	
4,000,000~–5,999,999	68 (29.6)	18 (22.8)	28 (39.4)	75 (30.1)	189 (30.0)	
6,000,000~–9,999,999	65 (28.3)	26 (32.9)	22 (31.0)	67 (26.9)	180 (28.6)	
~10,000,000	27 (11.7)	6 (7.6)	8 (11.3)	20 (8.0)	61 (9.7)	
Dinner preparation frequency						0.06
Never	30 (13.0)	21 (26.6)	15 (21.1)	51 (20.5)	106 (16.9)	
Sometimes	107 (46.5)	27 (34.2)	23 (32.4)	94 (37.8)	251 (39.9)	
Often	93 (40.4)	31 (39.2)	33 (46.5)	104 (41.8)	261 (41.5)	
Engaged in food education						0.92
Yes	23 (10.0)	8 (10.1)	6 (8.5)	21 (8.4)	58 (9.2)	
No	207 (90.0)	71 (89.9)	65 (91.5)	228 (91.6)	571 (90.8)	

*^a^Chi-square test*.

*Dinner preparing frequency was categorized by Never: never; Sometimes: less than once a month, a few times per month, 1–2 times per week; Often: 3–4 times per week, almost every day. Engaged in food education contains education regarding nutrition/cooking*.

### Differences in HEL and HEWSE Scores by Gender Role Group

**Figure 1:**
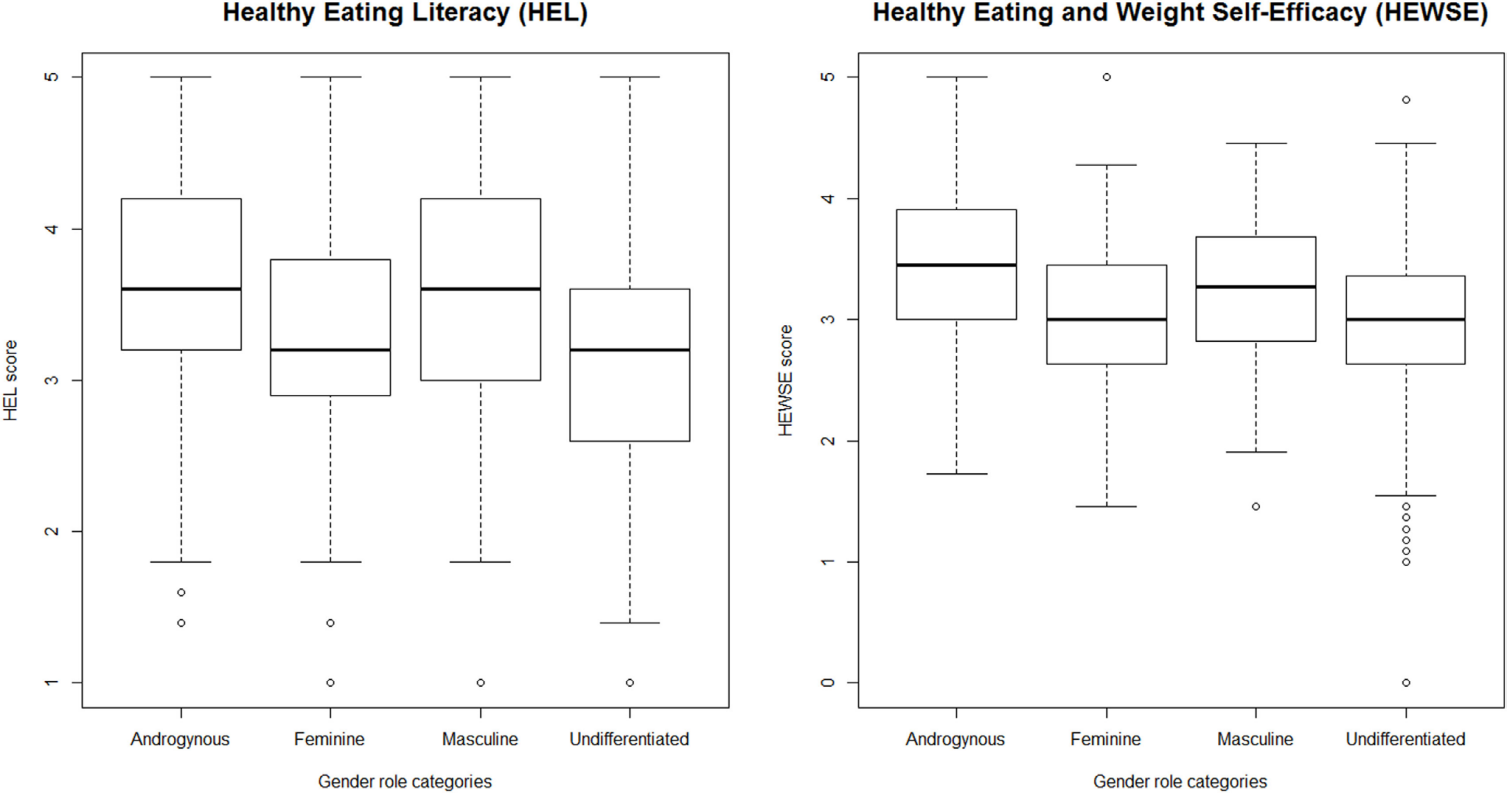
**Gender role differences in healthy eating literacy (HEL) and healthy eating and weight self-efficacy (HEWSE) scores**.

**Figure 2:**
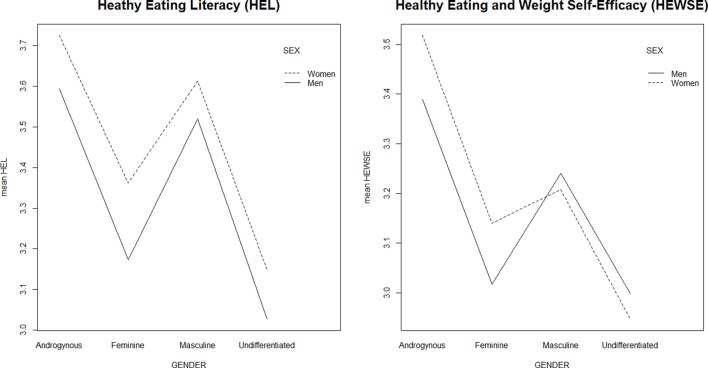
**Interaction between gender role orientation and biological sex in healthy eating literacy and healthy eating and weight self-efficacy scores**.

As shown in Table 3, there were statistically significant differences in the HEL [*F*(3, 625) = 25.41, *p* < 0.001] and HEWSE scores [*F*(3, 625) = 22.00, *p* < 0.001] for the gender role orientation categories. *Post hoc* tests showed that those in the Androgynous group had significantly higher HEL and HEWSE scores than those in the Feminine and Undifferentiated groups (Figure 1). In addition, the scores on both measures for the Masculine group were significantly higher than those for the Undifferentiated group. There was no interaction between gender role orientation and biological sex for the HEL [*F*(3, 621) = 0.06, *p* = 0.98] and the HEWSE [*F*(3, 621) = 0.93, *p* = 0.43] scales (Figure 2).

**Table 3:** **Analysis of variance for gender role differences in healthy eating literacy (HEL) and healthy eating and weight self-efficacy (HEWSE) scores**.

	Androgynous mean ± SD	Feminine mean ± SD	Masculine mean ± SD	Undifferentiated mean ± SD	*p*-value^a^	*Post hoc* Bonferroni
HEL	3.65 ± 0.71	3.27 ± 0.70	3.56 ± 0.81	3.10 ± 0.73	<0.001	A>F, A>U, M>U
HEWSE	3.45 ± 0.65	3.08 ± 0.64	3.23 ± 0.64	2.95 ± 0.71	<0.001	A>F, A>U, M>U

*^a^One-way ANOVA*.

### Multivariate Analyses

Table 4 shows the results of the hierarchical regression analyses of gender role orientation and food-related experiences on HEL and HEWSE scores, controlling for sociodemographic variables. In the first step of the hierarchical regression, sociodemographic variables and body perceptions were significant predictors only of HEWSE scores [*F*(5, 623) = 10.61, *p* < 0.001, *R*^2^ = 0.08]. Specifically, annual household income (β = 0.12, *p* < 0.01), and self-perceived weight status (β = 0.27, *p* < 0.001) were significant predictors of the HEWSE score. When gender role orientation was added in the second step of the hierarchical regression, the Androgynous and Masculine groups contributed significant unique variance in predicting the HEL [*F*(8, 620) = 9.23, *p* < 0.001, *R*^2^ = 0.11] and HEWSE scores [*F*(8, 620) = 14.79, *p* < 0.001, Δ*R*^2^ = 0.08, *R*^2^ = 0.17]. The Androgynous group (HEL β = 0.35, *p* < 0.001; HEWSE β = 0.32, *p* < 0.001) was a stronger predictor than the Masculine group in both scales (HEL β = 0.19, *p* < 0.001; HEWSE β = 0.10, *p* < 0.05). In the third step of the analysis, significant unique variance was only found in HEWSE scores [*F*(10, 618) = 15.51, *p* < 0.001, Δ*R*^2^ = 0.04, *R*^2^ = 0.20] for dinner preparation frequency (β = 0.16, *p* < 0.001) and having engaged in food education (β = 0.12, *p* < 0.01); gender role orientation remained significant after adding these variables (Androgynous: β = 0.30, *p* < 0.001; Masculine: β = 0.09, *p* < 0.05). For the HEL scores, biological sex was significant in the second step (β = 0.08, *p* < 0.05), but did not remain significant in the third step when Feminine became significant (β = 0.08, *p* < 0.05). On the basis of the hierarchical regression results, gender role orientation, especially the Androgynous group, better predicted individual HL and self-efficacy for healthy eating than the other predictors, including biological sex.

**Table 4:** **Hierarchical regression of sociodemographic factors, gender role orientation, and food experiences in the Healthy Eating Literacy and Healthy Eating and Weight Self-Efficacy scores**.

Step and variable	Step1			Step2			Step3		
	β	SE	*p*-value	β	SE	*p*-value	β	SE	*p*-value
**Healthy eating literacy**									
Sociodemographic									
Sex	0.047	0.042	0.26	0.082	0.040	0.038	0.060	0.041	0.14
Age	−0.054	0.041	0.19	−0.027	0.039	0.49	−0.029	0.039	0.46
Marital status	0.025	0.041	0.55	0.001	0.039	0.99	−0.006	0.039	0.87
Education	−0.002	0.042	0.95	0.010	0.039	0.80	0.015	0.039	0.70
Income	0.064	0.041	0.12	0.040	0.039	0.30	0.046	0.039	0.24
Body perception	0.017	0.040	0.67	−0.002	0.038	0.95	−0.001	0.038	0.98
Gender role orientation									
Androgynous				0.353	0.042	<0.001	0.344	0.042	<0.001
Feminine				0.078	0.041	0.056	0.081	0.041	0.047
Masculine				0.194	0.041	<0.001	0.191	0.041	<0.001
Food experiences									
Dinner preparation							0.065	0.040	0.11
Food education							0.063	0.038	0.10
F		1.07			9.23	<0.001		8.14	<0.001
Multiple R		0.010			0.12			0.13	
Multiple Adj R^2^		0.00070			0.11			0.11	
ΔR^2^					0.106	<0.001		0.010	0.052
**Healthy eating and weight self-efficacy scores**									
Sociodemographic									
Sex	−0.001	0.040	0.98	0.028	0.038	0.46	−0.024	0.039	0.54
Age	−0.021	0.039	0.59	0.003	0.038	0.93	−0.002	0.037	0.96
Marital status	−0.031	0.040	0.44	−0.050	0.038	0.19	−0.068	0.037	0.070
Education	−0.008	0.040	0.84	0.003	0.038	0.93	0.016	0.037	0.67
Income	0.124	0.039	0.0017	0.107	0.038	0.0047	0.119	0.037	0.0013
Body perception	0.269	0.038	<0.001	0.254	0.037	<0.001	0.256	0.036	<0.001
Gender role orientation									
Androgynous				0.320	0.041	<0.001	0.299	0.040	<0.001
Feminine				0.055	0.039	0.16	0.064	0.038	0.098
Masculine				0.099	0.039	0.012	0.092	0.038	0.0017
Food experiences									
Dinner preparation							0.162	0.038	<0.001
Food education							0.115	0.036	0.0014
F		10.61	<0.001		14.79	<0.001		15.51	<0.001
Multiple R		0.093			0.18			0.22	
Multiple Adj R^2^		0.084			0.17			0.20	
ΔR^2^					0.081	<0.001		0.038	<0.001

*Sex: women, 0; men, 1; Age: 25–29, 0; 30–34, 1; marital status: not married, 0; married, 1; education: less than University 0; University, 1; income: 1 (low)–4 (high); body perception: 1 (heavy), 2 (just about right), 3 (light); Androgynous: 0, 1; Feminine: 0, 1; Masculine: 0, 1; Food education:0.0, 1. Dinner preparing frequency was categorized by Never = 1: never; Sometimes = 2: less than once a month, a few times per month, 1–2 times per week; Often = 3: 3–4 times per week, almost every day*.

## Discussion

The present study explored the association between gender role orientation and HL and self-efficacy for healthy eating among Japanese in early adulthood. In the present study, majority of the participants were categorized as non-gender-typed personalities (i.e., androgynous and undifferentiated), which is consistent with a recent study in Japan (22). This may partly due to the recent changes in gender norms related to work and household tasks in Japan (24), and these traits are also seen in the United States. In the previous study, women’s BSRI masculine trait scores increased steadily over the 20-year period (13, 26), and the other study showed the significant decrease of women’s femininity scores between 1993 and 2012 (26). Gender personalities reflect the socio-cultural change, such as the second wave of the women’s movement (27), gendered-typed personalities seem to be gradually decreasing in modern society. Consistent with previous research, the results supported the hypothesis that people who are androgynous would have higher HL and self-efficacy for healthy eating. The results also confirmed that those in the Undifferentiated group had lower HL and self-efficacy. The effect of gender role was not diminished even after controlling for sociodemographic factors and food-related experiences. This suggests that individual gender role orientation may have a significant, unique influence on healthy eating. However, contrary to our expectation, there was no sex difference in HL and self-efficacy scores. Considering the significant difference in the frequency distribution of dinner preparation between men and women, Japanese men acquire HL and self-efficacy for healthy eating, but nonetheless, they do not seem to engage in everyday food preparation.

It is noteworthy that in addition to the Androgynous group, the Masculine group had high HL and self-efficacy scores. Contrary to our findings, masculinity has been suggested to play a role in risk behaviors not only for men but also sometimes for women (13). However, most previous studies were conducted in Western countries, and the effect of gender role orientation has not been studied in depth in Asian countries, including Japan. As gender role is deeply rooted in social and cultural context (28), our results might reflect current social expectations toward gender roles in Japan.

Food itself has a number of implicit meanings (29) and may be used to demonstrate an individual’s feelings, social status, identity, relationship with others, and so on (30). Food-related behaviors have also been widely used to demonstrate individual belief and create a particular impression on others (31), and this may also be applied to gender norms. For example, a recent study indicated that people who eat healthy diets and smaller meals have more feminine and less masculine images (31). Since 2008, the Japanese words “Soshoku Danshi (herbivore boys)” and “Nikushoku Jyoshi (carnivorous girls)” have been popular phrases, referring to men who are “feminine” instead of “masculine” (32), and vice versa for women, although it has nothing to do with what they eat. It is interesting that meat eating has long been tied to masculinity in social discourse (29, 30) and these concepts may have appeared with the recent arrival of gender-equal society in Japan. Our results showed that men and women in the Feminine and Undifferentiated groups had lower HL and self-efficacy for healthy eating, suggesting that low masculinity, irrespective of biological sex, might lead to less healthy eating beliefs and attitudes. Masculinity and femininity are associated with instrumental traits and expressive traits, respectively (8). For Japanese society, having high instrumental masculine traits appear to be related to healthy eating attitudes.

Individual gender role orientation and gender norms in society interact with each other. Social concepts of gender are produced and reproduced through underlying social norms of how people think and behave (33), and media representations (1). In the same way, an individual’s health behavior may be influenced by social norms (34). As Van Gundy and colleagues (28) observed, the effects of sex and gender role orientation on alcohol use vary by nation; their results indicated that sex-specific social expectations and responses were crucial for the prevention and treatment of health concerns across nations. Therefore, better understanding gender norms in the health of individuals may help to improve health at a population level (13). The findings of the present study may be relevant in promoting healthy eating from the perspective of gender norms, and now nutrition education should take gender perspective into them.

There are several limitations in the present study. First, homemakers were not included in the study sample, which might have affected women’s scores for HL and self-efficacy. In addition, because the sample consisted of white-collar workers and was recruited via an online survey company, the findings have limited generalizability. Second, because this was a cross-sectional study, the relationship between gender role orientation and health outcome was only observed at one point. However, gender role orientation and behavior patterns become relatively stable by the early adulthood (28). Therefore, an examination of the effect of the gender role orientation on an individual’s healthy eating in early adulthood may be assumed to apply throughout their adulthood. Third, outcomes were self-reported. We could not observe participants’ actual eating behaviors. Although HL and self-efficacy for healthy eating might be influenced by gender norms for healthy eating attitudes, actual food behaviors might be affected by environmental and practical factors, such as residence, family structure, and relationships. Also, we asked participants’ meal preparation frequencies, it may not be the appropriate measurements to investigate individuals’ healthy eating behaviors. Further study exploring the association between gender role orientation and objective eating behaviors is expected to determine actual health effects.

In summary, the present study showed that gender role orientation rather than biological sex had significant effects on HL and self-efficacy for healthy eating among Japanese in early adulthood. Directing attention to individual gender role orientation and gender norms in society may enhance more effective prevention and health education. Diverse approaches are necessary because gender norms in society are the result of continuous reproduction of many relationships, including family, couples, workplace, school, and the media. For example, research suggests that gender role development stems from the family context and may be influenced by parenting (35). The parents of men and women who are androgynous were reported to be less stereotyped in their definitions of gender roles, and offered a wider range of behavioral and attitudinal possibilities to their children (36). Applied to eating behaviors, a genderless home education in food preparation and cooking may be effective in reducing gender norms in food-related beliefs and attitudes. Making broader educational opportunity of nutrition for both sexes is also meaningful to reduce gender norms about food and gender, and to promote healthy eating habits. Health practitioners should work on providing nutrition advisement that is attractive for both sexes.

Overall, it is not pursuit of traditional gender role for each sex, but support of psychological development toward androgyny in both sexes that is important to promote healthy eating attitudes at the population level. In modern society, it is required that both sexes acquire the genderless behavioral traits to be able to manage their own dietary behaviors.

The present study, gender role orientation, was associated with HL and self-efficacy for healthy eating among Japanese in early adulthood, even after controlling for sociodemographic variables, including biological sex. The findings support those of previous research that people who are androgynous have better health practices. However, people in the Masculine group also had better HL and self-efficacy. There are still a limited number of studies considering gender norms as determinants of healthy eating, although numerous studies have explored “gender (biological sex)” differences in health. Further investigation of gender norms and eating behaviors would help improve individuals’ healthy eating.

## Author Contributions

CH conceived the research, collected the data, conducted statistical analyses, and drafted the manuscript; HI conceived the research, interpreted the results, and revised the manuscript; MO, MK, and TO interpreted the results; TK interpreted the results and supervised each process of the study; All authors read and approved the final manuscript.

## Conflict of Interest Statement

The authors declare that the research was conducted in the absence of any commercial or financial relationships that could be construed as a potential conflict of interest.
